# Ontology-aware neural network: a general framework for pattern mining from microbiome data

**DOI:** 10.1093/bib/bbac005

**Published:** 2022-01-29

**Authors:** Yuguo Zha, Kang Ning

**Affiliations:** Key Laboratory of Molecular Biophysics of the Ministry of Education, Hubei Key Laboratory of Bioinformatics and Molecular-imaging, Department of Bioinformatics and Systems Biology, Center of AI Biology, College of Life Science and Technology, Huazhong University of Science and Technology, 1037 Luoyu Road Wuhan, Hubei, Wuhan 430074, China; Key Laboratory of Molecular Biophysics of the Ministry of Education, Hubei Key Laboratory of Bioinformatics and Molecular-imaging, Department of Bioinformatics and Systems Biology, Center of AI Biology, College of Life Science and Technology, Huazhong University of Science and Technology, 1037 Luoyu Road Wuhan, Hubei, Wuhan 430074, China

**Keywords:** microbiome, pattern mining, ontology-aware, neural network, knowledge discovery

## Abstract

With the rapid accumulation of microbiome data around the world, numerous computational bioinformatics methods have been developed for pattern mining from such paramount microbiome data. Current microbiome data mining methods, such as gene and species mining, rely heavily on sequence comparison. Most of these methods, however, have a clear trade-off, particularly, when it comes to big-data analytical efficiency and accuracy. Microbiome entities are usually organized in ontology structures, and pattern mining methods that have considered ontology structures could offer advantages in mining efficiency and accuracy. Here, we have summarized the ontology-aware neural network (ONN) as a novel framework for microbiome data mining. We have discussed the applications of ONN in multiple contexts, including gene mining, species mining and microbial community dynamic pattern mining. We have then highlighted one of the most important characteristics of ONN, namely, novel knowledge discovery, which makes ONN a standout among all microbiome data mining methods. Finally, we have provided several applications to showcase the advantage of ONN over other methods in microbiome data mining. In summary, ONN represents a paradigm shift for pattern mining from microbiome data: from traditional machine learning approach to ontology-aware and model-based approach, which has found its broad application scenarios in microbiome data mining.

## Introduction

Pattern mining from microbiome data is a broad topic, which can include many types of knowledge to be discovered such as functional gene mining, novel species discovery, dynamic pattern mining and so on. Yet traditionally, microbiome data mining has heavily relied on sequence comparison for gene mining [1], species mining [2, 3] and taxonomic composition comparison for microbial community dynamic pattern discovery [4]. Machine learning methods have recently been used in a variety of microbiome data mining contexts whereas deep learning approaches are rarely developed in those contexts owing to the high heterogeneity of microbiome samples as well as the critical data involved in these pattern mining processes [5, 6].

**Figure 1:**
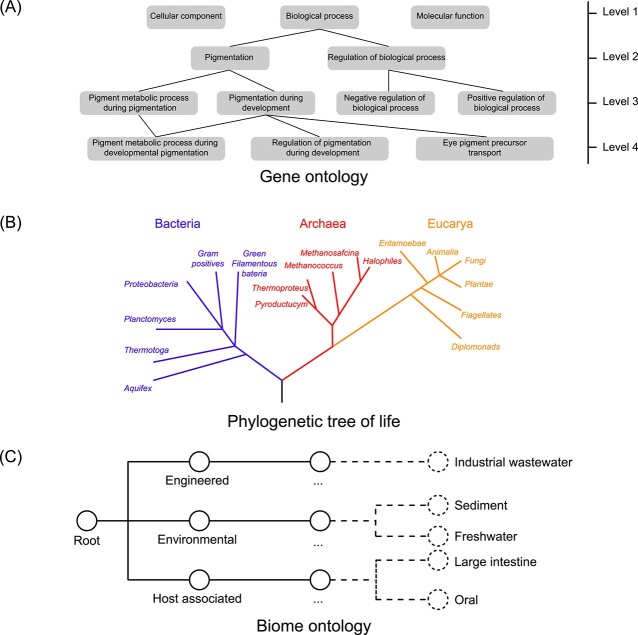
Microbiome entities are usually organized in an ontology structure. (**A**) Functional genes are organized according to general ontology such as gene ontology. (**B**) Species are organized according to the phylogenetic tree of life. (**C**) Microbial community samples are organized according to the biome ontology.

Microbiome entities are usually organized in ontology structures (Figure 1). Functional genes are organized using general ontology such as Gene Ontology (GO) [7] or specialized ontology such as the antibiotic resistance ontology [8]. Species are organized using the phylogenetic tree of life [9, 10], and microbial community samples are organized using the biome ontology [11]. The structure of ontology can be described in terms of a directed graph, wherein each term is a node, and the relationships among the terms are directed edges between the nodes, representing the hierarchical relationships of two terms: usually the edge source term contains the edge target term. Specifically, all the defining terms are organized by a structured hierarchy, which is called the ontology structure. For example, in the antibiotic resistance ontology, the term ‘non–beta-lactam’ is parent of the term ‘aminoglycoside’. Therefore, there is a directed edge between ‘non–beta-lactam’ and ‘aminoglycoside’. Recently, pattern mining methods that have considered ontology structures have shown the advantages to be gained in mining efficiency and accuracy [12–15]. However, the neural network represents an advanced approach for microbiome data mining [16–19]. Therefore, building neural network models together with ontology structures of microbiome entities should facilitate pattern mining from microbiome data.

In this work, we have summarized the ontology-aware neural network (ONN) as a general framework for microbiome data mining. We first introduce the ONN approach as a general framework for pattern mining from microbiome data, for a broad spectrum of microbiome data mining scenarios. Then, we discuss about applications of ONN in multiple contexts, including gene mining, species mining and dynamic pattern mining. We then highlighted one of the most important characteristics of ONN, namely novel knowledge discovery, which makes ONN a standout among all microbiome data mining methods. Taken together, ONN represents a paradigm shift for pattern mining from microbiome data: from traditional machine learning approach to ontology-aware and model-based approach, which has found its broad application scenarios in microbiome data mining.

## Current methods and deep learning for microbiome data mining

There are already computational solutions for pattern mining from microbiome data [20–23] (Table 1). Most of these methods, however, have a clear trade-off, particularly when it comes to big-data analytical efficiency and accuracy.

**Table 1:** Current methods and deep learning for microbiome data mining

**Category**	**Traditional methods**	**Deep learning methods**	**Description**
ARG mining	ResFinder [1]	ONN4ARG [13], DeepARG [16]	Deep learning methods could identify novel ARG with high efficiency
BGC mining	AntiSMASH [25], ClusterFinder [26]	DeepBGC [27]	AI methods are suitable for detection of BGCs of known classes from bacterial genomes
Microbial source tracking	SourceTracker [5], FEAST [6]	ONN4MST [14], EXPERT [15]	Deep learning methods are especially suitable for source tracking among thousands to millions of samples in a fast and accurate manner

*Note*: AI, artificial intelligence; BGC, biosynthetic gene cluster.

Antibiotic resistance gene (ARG) is a major challenge for microbiome data mining, which aims to predict the presence of ARG from metagenomic data in livestock manure, compost, wastewater treatment plants, soil, water and other affected environments as well as within the human microbiome. Traditional methods for the identification of ARG are based on the computational principle of comparison of the metagenomic DNA sequences against available online databases (e.g. CARD [8]). Such comparison is performed by aligning raw reads or predicted open reading frames (full–gene-length sequences) from the assembled contigs to the database of choice, using programs such as BLAST, Diamond [24] and so on. However, traditional methods are limited to identifying ARGs that are close homologous genes to known ARGs in the database and cannot identify remote homologous genes or novel ARGs. Recently, several deep learning solutions have been proposed for ARG prediction. First is DeepARG [16], which is based on a deep neural network model and the second is HMD-ARG [17], which conducts a convolutional neural network model. The input of deep learning approaches can be bit-score (for DeepARG) or one-hot encoding vector of protein sequence (for HMD-ARG). Deep learning, unlike traditional sequence alignment methods, leads to model-based methods that can quickly profile ARGs in large-scale metagenomic data and predict ARGs from billions of candidates [16, 17].

Functional gene mining not only focuses on single gene identification such as ARG but also attempts to predict a set of functional genes, that is a biosynthetic gene cluster (BGC). Natural products represent a rich reservoir of small molecule drug candidates. These molecules are microbial secondary metabolites synthesized by co-localized genes termed BGC. Numerous bioinformatics tools [25–27] have leveraged the increasingly abundant genomic data to facilitate BGC mining. Early approaches implemented simple BGC reference alignment techniques using programs such as BLAST and were often paired with manual curation. ClusterFinder makes use of a Hidden Markov Model to improve the ability to find new BGC genomic elements [26]. DeepBGC is a recently released deep learning solution that uses a bidirectional long-/short-term memory recurrent neural network model to improve detection of BGCs of known classes from bacterial genomes and has the potential to detect novel classes of BGCs [27].

Microorganisms can be found in almost every environment of the Earth’s biosphere and are responsible for numerous biological activities including carbon and nitrogen cycling [28], soil organic matter [29] and human health and disease [30]. Phylogenetic analyses of these microorganisms have revealed that the composition of human gut microbiomes is affected by the host [31], while additional research has illustrated dynamic changes of gut microbiota in the adaptation to the host [32]. It is critical to identify and characterize microbial species in environments and individual human hosts in order to learn about human–microbial interactions. Many bioinformatics computational tools have been developed for the characterization and identification of microorganisms at species or strain levels, such as StrainPhlAn [33], ConStrains [34] and Strain-GeMS [2]. However, most of these traditional tools are based on genomic sequence comparison and marker genes such as 16S rRNA and thus often lack the resolution to reliably capture intraspecific genomic differences.

Microbial source tracking also remains challenging for microbiome data mining, which aims to estimate the proportion of contaminants in a given community that come from possible source environments. Many methods have been proposed to accurately estimate the contribution of hundreds of potential source environments for a community sample promptly. For example, the Bayesian-based method SourceTracker [5] and expectation–maximization-based method FEAST [6] could achieve high accuracy regarding hundreds of microbial community samples from a handful of biomes. However, the time cost of source tracking would increase rapidly as the number of samples and biomes increases, preventing these methods from large-scale microbial source tracking. Notably, large-scale microbial source tracking is of vital importance because it can help researchers quickly identify all possible sources of samples and narrow the tracing range, which has great potential in some applications, such as forensic studies [35]. Deep learning solutions, such as ONN4MST [14] and EXPERT [15], have recently been proposed to solve this problem. Model-based methods, such as the neural network, are used in these deep learning solutions to model microbial community structures, and the speed and accuracy of the source tracking procedure could be greatly improved.

Current methods for microbiome data mining have a trade-off between big-data analytical efficiency and accuracy. Deep learning methods eliminate this trade-off. Those deep learning methods take advantage of deep learning models (i.e. neural network), and both accuracy and efficiency could be largely improved compared with traditional methods. For example, Kang *et al*. reported an ultrafast and interpretable source tracking method (i.e. ONN4MST), which utilizes a novel deep learning model to profile microbial community structures [14]. ONN4MST achieved the prediction accuracy of 0.97 when faced with millions of samples from thousands of biomes, while the running time of the entire source tracking procedure could be within 1 s per sample. Deep learning methods are advantageous in the generation of the models from a massive amount of samples, which are representative of the global profile of the context-dependent subjects [36]. Deep learning methods are therefore suitable for accurate and fast search when new samples (either a gene, species or community) are searched against models [37–39]. We summarized current methods and deep learning methods for microbiome data mining in Table 1.

**Figure 2:**
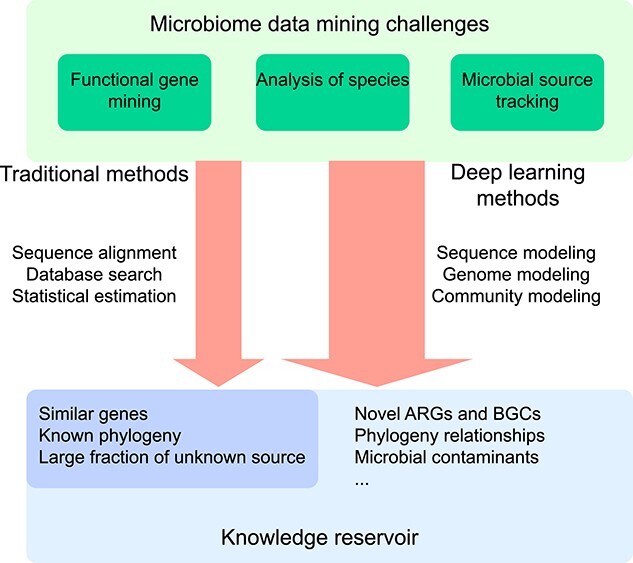
Advantages of deep learning methods for pattern mining from microbiome data. Most of current methods have a trade-off between big-data analytical efficiency and accuracy, for pattern mining from microbiome data. Compared with traditional methods, deep learning methods modeling microbiome data and thus has potential to discover novel knowledge. Traditional methods usually take sequence alignment, database search and statistical estimation for pattern mining from microbiome data. Deep learning methods take modeling approaches and thus could lead to more comprehensive knowledge discovery.

In summary, deep learning methods are particularly suitable for solving the problem of source tracking, gene mining and other patterns mining. The advantages of deep learning methods for pattern mining from microbiome data are shown in Figure 2.

## Onn as a general framework for pattern mining from microbiome data

**Figure 3:**
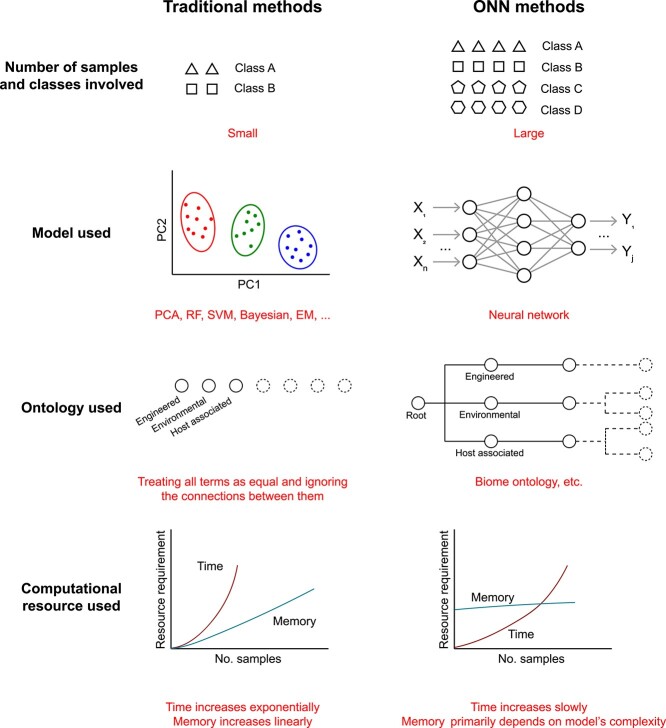
The differences between traditional methods and ONN for microbiome data mining. ONN is suitable for large-scale samples pattern mining from microbiome data. The model used by traditional methods are usually machine learning models, including principle component analysis, random forest, supporting vector machine, Bayesian, and expectation–maximization. ONN employs advanced models such as neural network. ONN utilizes ontology information and thus are able to identify genes, species, and patterns hierarchically, facilitating knowledge discovery from multiple dimensions. The trade-off between big-data analytical efficiency and accuracy of current methods for microbiome data mining can be solved by ONN methods.

Microbiome entities are usually organized in ontology structures (Figure 1), which inspires us to seek a general deep learning framework, that is compatible with the ontology structure of microbiome data. Here, we summarized ONN as a general framework for pattern mining from microbiome data. The advantages of ONN are established from several aspects (Figure 3). First, ONN is suitable for large-scale sample pattern mining from microbiome data. Second, ONN employs an advanced deep learning model (i.e. neural network), which has shown superiority in many fields of microbiome data mining. Third, ONN utilizes ontology information and thus can identify genes, species and patterns hierarchically, thus facilitating knowledge discovery from multiple dimensions. Moreover, ONN eliminates the trade-off between big-data’s analytical efficiency and accuracy of current methods for microbiome data mining.

The ontology structure of microbiome entities (e.g. gene ontology, biome ontology) and the neural network model adapted to specific problems are two key components of ONN. ONN was able to understand the hierarchy of microbiome entities by incorporating ontology structure into neural network models. ONN uses a novel ontology-aware layer to implement the model, which encodes the ontology information. As a result, ONN generates hierarchical annotations according to the ontology used. ONN can be easily applied to any microbiome data mining problem involving ontology structures, such as gene mining, species mining and dynamic pattern mining.

### Gene mining

With the advanced sequencing technology and development of microbiome culture strategies, many microbiome projects focusing on different biomes have been proposed: for example, the Human Microbiome project for sequencing human gut microbiome [21], Tara Oceans project [40] for sequencing global ocean microbiome and Earth Microbiome project [22] for sequencing global soil microbiome. These projects have provided a large number of microbial genomes, which provide big reservoirs of functional genes. However, the functional diversity of microbiomes has not been fully explored, and about 40% of microbial gene functions remains to be discovered [41]. ARG represents one special category of functional genes, which is an urgent and growing threat to public health. The discovery of resistance genes in diverse environments offers possibilities for early surveillance, actions to reduce transmission, gene-based diagnostics and, ultimately, improved treatment. Currently, numerous ARG databases and ARG predictive tools have been established or proposed. For example, the comprehensive ARG database, i.e. CARD [8] is the most used ARG database. CARD is a rigorously curated collection of known resistance determinants and associated antibiotics, organized by the antibiotic resistance ontology that organizes ARGs according to their corresponding drug classes.

### Species mining

Traditional microbiome studies have primarily focused on bacteria although bacteria only represent a small fraction of all microorganisms. In addition to bacteria, archaea, viruses, and protists are also often abundant in environments. Archaea are generally dominant in extreme environments and define the limits of life on Earth in many cases [42]. Archaea were originally discovered and described in extreme environments including in high salinity [43], extremely acidic [44] and anerobic environments [45]. Viruses, as very small infectious agents, rely on living cells to multiply and are the smallest and most abundant of all microorganisms [46]. Protists are unicellular eukaryotic microorganisms that exhibit less complex physiological structures than other eukaryotes. Although microorganisms harbor very important functional genes, most of their genomic contents remains poorly understood. For example, over 60 000 protist species have been identified in the NCBI (National Center for Biotechnology Information) taxonomy system, while many have also yet to be identified [47].

### Dynamic pattern mining

Niche-specific spatiotemporal dynamics within microbial communities in addition to the consequences of these spatiotemporal dynamics on species evolution are key determinants for the formation, development, stability and dynamics of microbial communities [48]. However, many microbial ecological and evolutionary patterns remain to be discovered: for example, the temporal dynamics of human gut microbial communities. Human gut microbiota rapidly respond to changes in diet [49, 50], and the composition of an individual’s gut microbiota is predominantly determined by dietary habits over the long term (i.e. >1 year) [51, 52]. However, these dynamics are highly variable among individuals [53]. Over short-term time scales (i.e. <1 month), human gut microbiota can drastically change during dietary shifts, while such changes can also be quickly reversed after shifts in diet [4]. In addition, strong plastic patterns can be observed over mid-term time scales (i.e. between a month and a year). Overall, investigations into these problems could help develop a better understanding of the ecological and evolutionary patterns ranging from small to large scales.

### Disease pattern mining

Increasing evidence suggests that the human microbiome, not just the gut microbiome, is tightly related to a variety of diseases, including chronic diseases [54], inflammation diseases [55] and cancer [30]. Revealing the relationships between human diseases and microbes can not only promote our understanding of the disease pathogenesis but also provide new strategies for the diagnosis and treatment of diseases. Multiple computational models have been developed in recent years to predict microbes that are linked to diseases. These computational models include a wide range of algorithms and models for analyzing microbiome data, such as score-function-based models, network algorithm-based models, machine learning-based models and experimental analysis-based models [56]. The relationship between the human microbiome and specific diseases, however, is far from clear, let alone the intricate patterns that could be used to differentiate these diseases [57]. Bottlenecks can occur as a result of batch effects between multiple cohorts [58], the dynamic nature of diseases [59] and so forth. Traditional machine learning methods are unable to distinguish diseases based on the human microbiome despite the fact that some diseases, such as inflammatory bowel disease, may share a high proportion of microbes [60].

## Applications of onn in microbiome data mining contexts

Recently, a series of ONN methods have been developed for pattern mining from microbiome data. Those ONN methods have achieved robust performance compared with traditional machine learning methods or other deep learning methods that do not consider biological ontology. In this section, we summarize several major applications of ONN in microbiome data mining contexts (Table 2).

**Table 2:** The application of ONN in multiple contexts

**Method**	**Category**	**Description**	**Reference**
DeepGO	Gene function prediction	DeepGO utilizes the dependencies between GO classes as background information to construct an ONN model and specifically outputs information in the hierarchical structure of the GO	[12]
ONN4ARG	Functional gene prediction	ONN4ARG was proposed to solve problems in novel ARG identification and make efforts for comprehensive profiling of ARGs in diverse environments. ONN4ARG is an ONN model that employs a novel ontology-aware layer and generates multilevel annotations of antibiotic resistance types	[13]
ONN4MST	Dynamic pattern mining	ONN4MST was proposed for microbial source tracking. The ONN model can utilize the biome ontology information to model the dependencies between biomes, and estimate the proportion of various biomes in a community sample	[14]
EXPERT	Disease prediction	EXPERT is an exact and pervasive expert model for source tracking microbial communities based on transfer learning. EXPERT could easily expand the supervised model’s search scope to include the context-dependent community samples and understudied biomes (e.g. samples from different disease stages)	[15]

### Gene function predicting and ARG mining

A large number of protein sequences are becoming available through the application of novel high-throughput sequencing technologies. Experimental functional characterization of these proteins is time consuming and expensive and is often only done rigorously for a few selected model organisms. Computational function prediction approaches have been suggested to fill this gap. The functions of proteins are classified using the GO, which contains over 40 000 classes. To address the problem in protein function prediction, DeepGO [12] utilizes the dependencies between GO classes as background information to construct an ONN model and specifically outputs information in the hierarchical structure of the GO. Developers compared DeepGO with the other two top-performing methods on a standard benchmark data set, and results show DeepGO achieved the highest area under the curve (AUC) of 88%.

ARG represents a specific class of functional genes, which enable bacteria to survive under extremely antibiotic environments. The discovery of resistance genes in diverse environments offers possibilities for early surveillance, actions to reduce transmission, gene-based diagnostics, and, ultimately, improved treatment. Recently, ONN4ARG [13] has been proposed to solve problems in novel ARG identification and make efforts for comprehensive profiling of ARGs in diverse environments. ONN4ARG is an ONN model that employs a novel ontology-aware layer and generates multilevel annotations of antibiotic resistance types. Systematic evaluations show that the ONN4ARG model has profound performance improvement over state-of-the-art models such as DeepARG [16], especially for the detection of remotely homologous ARGs. Experiments based on more than 200 million candidate microbial genes collected from thousands of samples from various environments have resulted in submillion candidate ARGs and more than 40 000 putative novel ARGs, which have greatly expanded existing ARG repositories [16]. Furthermore, we compared the ONN method (i.e. ONN4ARG) with current standard methods for ARG predicting. The ONN model of ONN4ARG is built based on CARD version 3.0.3. When we compared the latest CARD version 3.1.4 with the previous version 3.0.3, we discovered 2281 new ARGs. The 2281 ARGs were then clustered into 312 clusters with a 90% sequence identity, and the 312 representative ARGs were used as the testing data set. For Diamond, we searched CARD version 3.0.3 with the testing data set. For DeepARG, we used the DeepARG program (default parameters) to predict the testing data set. For ONN4ARG, we used the ONN4ARG program (default parameters) to predict the testing data set. The ONN4ARG method outperformed current ARG prediction methods (Diamond and DeepARG) in terms of accuracy and efficiency, i.e. high accuracy and less time required given that the memory usage is acceptable for a regular laptop computer (Supplementary Table S1 available online at http://bib.oxfordjournals.org/). ONN4ARG achieved better performance than DeepARG largely because it was able to identify remote homologous ARGs. For example, ONN4ARG predicted one representative ARG (WP_122630831.1) in the testing data set to be an ARG, but DeepARG predicted it to be non-ARG. Notably, the representative ARG (WP_122630831.1) shares a remotely sequence identity of 48.9% with its closest homologous ARG (CAQ53840.1) in DeepARG database (i.e. ARGminer v1.1.1). We also searched WP_122630831.1 against the CARD version 3.0.3, and the closest homologous ARG in CARD version 3.0.3 is also CAQ53840.1. Therefore, results on this representative ARG (WP_122630831.1) have confirmed that ONN4ARG could be superior than other methods in the discovery of novel ARGs.

### Species mining, including bacteria, virus and protist mining

Despite the fact that microorganisms contain many important functional genes, the majority of their genomic content is still unknown. In the NCBI taxonomy system, for example, over 60 000 protist species have been identified, with many more yet to be identified [46]. Numerous bioinformatics tools have taken advantage of the growing amount of genomic data to identify new species. For example, StrainPhlAn [33], ConStrains [34] and Strain-GeMS [2] are proposed for bacterial identification at the strain level based on genomic information and ArboTyping [61] for the identification of virus species and genotypes. By considering that species are organized according to the phylogenetic tree of life, the latter can be considered an ontology structure, and the identification of novel species is an ontology-related problem. As a result, ONN is appropriate for species mining.

### Microbial source tracking

With the rapid accumulation of microbial community samples from various niches (also referred to as biomes) around the world, knowledge about microbial communities and their influence on the environment and human health has grown rapidly. The rapid accumulation of microbial community samples has provided the opportunity to investigate the interactions among microbes, human health and the environment. Those community samples have created an enormous hurdle for characterizing the potential inputs from other associated biomes, thus calling for fast and accurate microbial source tracking. Considerable attention has been paid to exploring the interactions on small scales, such as disease diagnosis, early development, pregnancy and immigration whereas integrative, large-scale and scalable investigations are understudied. Such investigations are challenging for a few reasons. First, the number of samples easily exceeds millions whereas the number of niches exceeds hundreds, and microbial source tracking becomes a very complex task. Second, the noises that exist in the rich-sourced data might hire important patterns invisible for traditional methods. To address these limitations, ONN4MST was proposed for microbial source tracking. The ONN4MST model employs a novel ontology-aware approach that encourages prediction satisfying the ‘biome ontology’. In other words, the ONN model can utilize the biome ontology information to model the dependencies between biomes and estimate the proportion of various biomes in a community sample. ONN4MST has provided an ultrafast (<0.1 s) and accurate (AUC >0.97 in most cases) solution for searching a sample against the data set containing hundreds of potential biomes and millions of samples, and also outperformed state-of-the-art methods in scalability and stability. Furthermore, we compared ONN method (i.e. ONN4MST) with current standard methods for microbial source tracking. The data set used for evaluation consists of 10 270 microbial community samples selected from the data sets used in published study by Shenhav *et al*. [6]. In terms of accuracy and efficiency, the ONN4MST method outperformed current standard microbial source tracking methods (SourceTracker and FEAST), with a higher Area Under the Receiver Operating Characteristic curve (AUROC) and a significant reduction in time usage. (Supplementary Table S2 available online at http://bib.oxfordjournals.org/). Links to the testing data set are provided in Supplementary Table S3 available online at http://bib.oxfordjournals.org/.

### Disease prediction

Microorganisms have been discovered to be closely related to a variety of important human diseases. The growing number of human microbe–disease associations provides important insights into the underlying disease mechanism from the perspective of human microbes, which is extremely useful for pathogenesis research, early diagnosis and precision medicine [62, 63]. EXPERT [15] is an exact and pervasive expert model for source tracking microbial communities based on transfer learning. Built on the ontology information and transfer learning techniques, EXPERT has acquired the context-aware flexibility and could easily expand the supervised model’s search scope to include the context-dependent community samples and understudied biomes. EXPERT considers gut communities at different disease stages as context-dependent biomes and estimates the contribution from different disease stages for a given microbial community sample. EXPERT’s utility in characterizing human gut microbial communities associated with different types of diseases is superior to current standard methods. The superiority of EXPERT has been demonstrated in disease pattern mining. For example, when dealing with 635 samples from a recent study of colorectal cancer, EXPERT could achieve an AUROC of 98% when predicting the host’s phenotypical status [15].

To sum up, we have demonstrated that ONN could be applied on a broad spectrum of applications, including functional gene mining, novel species mining and microbial community dynamic pattern mining. Of note, ONN is especially suitable for disease pattern mining, which is very robust against batch effects and other confounding factors.

## Onn modeling tells us more about unknown than known

The deep learning approach could best utilize the ontology information hidden from the biological big data. At the lowest ontology level, the deep learning approach should be comparable with other methods. However, on the higher ontology levels, the deep learning approach could identify remote similarities among genes, species and patterns of interest.

**Figure 4:**
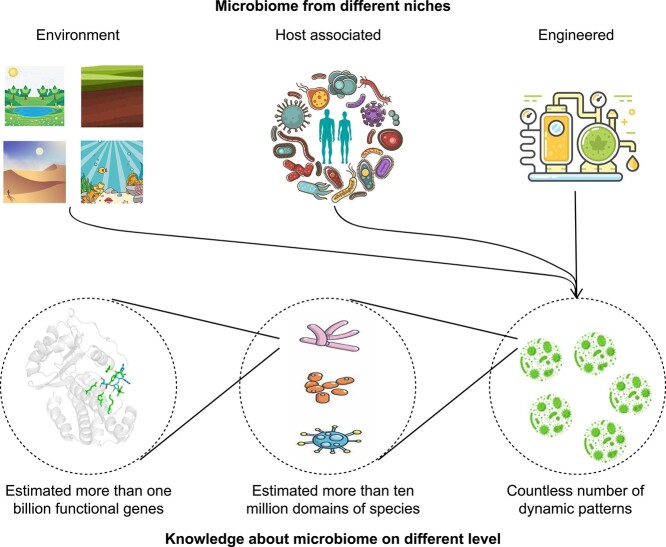
The hierarchical organization of knowledge about microbiome. Microbiome samples could be collected from diverse niches around the world, including environment (e.g. soil, water and air), host associated (e.g. human gut, oral and skin), and engineered (e.g. fermentation). Genes, species and communities represent the three level of microbiome knowledge. The ontological organization of the knowledge about microbiome, whether on gene, species or community levels, could naturally lead us to the discovery of new knowledge about microbiome.

Microbiome samples could be collected from diverse niches around the world, and genes, species and communities represent the three levels of microbiome knowledge. The ontological organization of the knowledge about microbiomes, whether on gene, species or community levels, could naturally lead us to the discovery of new knowledge about microbiomes (Figure 4). Currently, we know little at all of the gene, species and community levels, and we can only obtain more knowledge if the expanding of our knowledge could keep pace with the increasing changes at these levels.

Compared with traditional methods, ONN is not advantageous for the identification of genes, species and communities in existing databases as numerous methods already exist for database searches, sequence comparisons and structure comparisons. However, ONN’s performance is equally good compared with existing methods proving the power of neural network models on such data mining processes.

**Figure 5:**
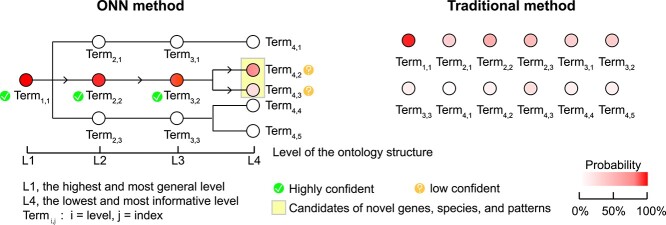
Rationale on ‘why’ ONN could discover novel knowledge from microbiome data. Left: ONN outputs hierarchical predictions with predicted probability scores. In most cases, ONN makes highly confident predictions at higher levels (e.g. L1, L2 and L3 in the figure) and low confirmatory predictions at lower levels (e.g. L4 in the figure). Those low confirmatory predictions are potential candidates of novel genes, species and patterns. ONN can give information about ancestors (or categories at higher levels) of those potential candidates and provide clues about novel knowledge. Right: In contrast, traditional methods without considering ontology treat all terms as equal, so that no candidate (at lower levels of the hierarchical structure) has highly confident prediction, resulting in no clue about the potentially novel knowledge. ONN showed its advantages on the discovery of novel genes, species and communities, largely due to its ability to sense the ontology structure, and leads to the discovery at higher levels of the ontology structure.

ONN demonstrated its advantages in the discovery of novel genes, species and communities owing to its ability to sense the ontology structure and lead to discovery at higher levels of the ontology structure (Figure 5). ONN outputs hierarchical predictions with predicted probability scores. In most cases, ONN makes confident predictions at higher levels and less confirmatory predictions at lower levels. Those less confirmatory predictions are potential candidates for novel genes, species and patterns. ONN can give information about ancestors (or categories at higher levels) of those potential candidates, which is impracticable for methods without considering ontology. One example is on functional gene discovery: GAR is a newly discovered non–beta-lactam aminoglycoside resistance gene (e.g. gentamicin, micronomicin), that is not found in any existing databases [13]. With both DeepARG and HMD-ARG models, search results show that the GAR is not an actual ARG. ONN4ARG, however, correctly identified GAR as an ARG resistant to non–beta-lactam antibiotics. Despite the fact that ONN4ARG could only predict GAR as a non–beta-lactam rather than a subtype of aminoglycoside, it was the only method used in this study that could predict GAR as an ARG demonstrating ONN4ARG’s capability for knowledge discovery [13]. Another example is on microbial community sample source tracking: a microbial source tracking investigation that involved 11 microbial community samples from groundwater biome also showed the capability of ONN4MST for knowledge discovery [14]. ONN4MST could identify a large proportion of these groundwater samples from aquatic biomes coupled with a considerable proportion from terrestrial biome, thus suggesting that the samples might be collected from terrestrial water (i.e. river, lake and groundwater) or its sediment. In contrast, FEAST assigned a large proportion of unknowns for these groundwater samples.

In summary, ONN could enable novel knowledge discovery from microbiome data at multiple levels: gene, species, communities and so forth. These can deepen our understanding of how microbial communities are assembled and functioned, leading to better utilization of microbiome data in environmental and clinical applications.

## Conclusions

ONN is a general framework that can be used for a broad spectrum of microbiome data mining applications. On the one hand, in many contexts, the biological data are organized in the hierarchical or ontological manner; therefore, ONN is naturally suitable for these types of data. On the other hand, neural network methods could generate models that always outperform traditional methods in gene mining, species mining and dynamic pattern discovery. Therefore, ONN, which combines ontology awareness and neural network models, could greatly facilitate pattern mining from microbiome data.

ONN has revealed an excellent pool of knowledge about microbiome patterns at gene, species and community levels, and it would lead to broader and deeper knowledge about microbiomes. The power of ONN on knowledge discovery has been exemplified in many contexts: whether novel genes, new species or novel dynamic patterns of communities, ONNs have already led us to the discovery of new knowledge about microbiomes whereas there might still be a large space toward the full picture about the microbiome worlds.

It should be noted that the ONN method could be powerful but might not be a general and all-purpose method. Its usefulness in the context of functional gene mining and microbial source tracking have been proved in previous work and benchmarked in this work, yet its applicability in novel species mining has not been proved yet. Thus, though ONN could be a general framework for pattern mining from microbiome data, its utility in the broad spectrum of microbiome data mining applications should be worthy of further investigation.

There is no doubt that ONN has limitations. First of all, a good ontology structure is always dependent on domain knowledge, which is not readily available in numerous contextualized applications. The ONN model is also limited by the interpretability of its results: the quantitative classification results could be used for gene mining or sample source tracking whereas the exact accuracy of such pattern mining remains to be determined. Furthermore, ONN’s robustness against batch effects, as well as its applicability in contextualized applications that have very few samples, remains to be examined. Despite these limitations which remain to be overcome, ONN represents a paradigm shift for pattern mining from microbiome data: from traditional machine learning approach to ontology-aware and model-based approach, which has found its broad application scenarios in microbiome data mining.

Finally, we should admit that currently we know little at all of the gene, species and community levels, and we can only obtain more knowledge if the expanding of our knowledge could keep pace with the increasing changes at these levels. ONN is a powerful tool toward this end, and we hope similar ideas and methods could be used in the broad spectrum to speed up the knowledge discovery in the microbial world.

**Key Points**

- Microbiome entities are usually organized in ontology structure, and pattern mining methods consider ontology structures.
- ONN, which has considered ontology structures, could offer advantages in mining efficiency and accuracy for microbiome data mining.
- ONN could be used in multiple contexts, including gene mining, species mining and microbial community dynamic pattern mining.
- ONN could discover novel knowledge from microbiome data, thus making it a standout among all microbiome data mining methods.

## Supplementary Material

Supplementary_Table_S1_bbac005

Supplementary_Table_S2_bbac005

Supplementary_Table_S3_bbac005

## Notes

**Yuguo Zha** is a PhD student at the Huazhong University of Science and Technology. His research interests are in microbiome-associated data mining, including gene mining, species mining and pattern mining.

**Kang Ning** is a professor at the Department of Bioinformatics and Systems Biology, College of Life Science and Technology, Huazhong University of Science and Technology.

## References

[1] Bortolaia V, Kaas RS, Ruppe E, et al. ResFinder 4.0 for predictions of phenotypes from genotypes. J Antimicrob Chemother 2020;75:3491–500. 32780112 10.1093/jac/dkaa345 PMC7662176

[2] Tan C, Cui W, Cui X, et al. Strain-GeMS: optimized subspecies identification from microbiome data based on accurate variant modeling. Bioinformatics 2019;35:1789–91. 30295697 10.1093/bioinformatics/bty844

[3] Tu Q, He Z, Zhou J. Strain/species identification in metagenomes using genome-specific markers. Nucleic Acids Res 2014;42:e67–7. 24523352 10.1093/nar/gku138 PMC4005670

[4] Liu H, Han M, Li SC, et al. Resilience of human gut microbial communities for the long stay with multiple dietary shifts. Gut 2019;68:2254. 30420399 10.1136/gutjnl-2018-317298 PMC6872438

[5] Knights D, Kuczynski J, Charlson ES, et al. Bayesian community-wide culture-independent microbial source tracking. Nat Methods 2011;8:761–3. 21765408 10.1038/nmeth.1650 PMC3791591

[6] Shenhav L, Thompson M, Joseph TA, et al. FEAST: fast expectation-maximization for microbial source tracking. Nat Methods 2019;16:627–32. 31182859 10.1038/s41592-019-0431-x PMC8535041

[7] The Gene Ontology Consortium . The Gene Ontology resource: enriching a GOld mine. Nucleic Acids Res 2021;49:D325–34. 33290552 10.1093/nar/gkaa1113 PMC7779012

[8] Alcock BP, Raphenya AR, Lau TTY, et al. CARD 2020: antibiotic resistome surveillance with the comprehensive antibiotic resistance database. Nucleic Acids Res 2020;48:D517–25. 31665441 10.1093/nar/gkz935 PMC7145624

[9] Hinchliff CE, Smith SA, Allman JF, et al. Synthesis of phylogeny and taxonomy into a comprehensive tree of life. Proc Natl Acad Sci U S A 2015;112:12764. 26385966 10.1073/pnas.1423041112 PMC4611642

[10] Hug LA, Baker BJ, Anantharaman K, et al. A new view of the tree of life. Nat Microbiol 2016;1:16048. 27572647 10.1038/nmicrobiol.2016.48

[11] Mitchell AL, Almeida A, Beracochea M, et al. MGnify: the microbiome analysis resource in 2020. Nucleic Acids Res 2020;48:D570–8. 31696235 10.1093/nar/gkz1035 PMC7145632

[12] Kulmanov M, Khan MA, Hoehndorf R. DeepGO: predicting protein functions from sequence and interactions using a deep ontology-aware classifier. Bioinformatics 2018;34:660–8. 29028931 10.1093/bioinformatics/btx624 PMC5860606

[13] Zha Y, Chen C, Jiao Q, et al. Ontology-aware deep learning enables novel antibiotic resistance gene discovery towards comprehensive profiling of ARGs. bioRxiv 2021; 2021.2007.2030.454403.

[14] Zha Y, Chong H, Qiu H, et al. Ontology-aware deep learning enables ultrafast, accurate and interpretable source tracking among sub-million microbial community samples from hundreds of niches. bioRxiv 2020; 2020.2011.2001.364208. 10.1186/s13073-022-01047-5 PMC9040266 35473941

[15] Chong H, Yu Q, Zha Y, et al. Enabling technology for microbial source tracking based on transfer learning: from ontology-aware general knowledge to context-aware expert systems. bioRxiv 2021; 2021.2001.2029.428751.

[16] Arango-Argoty G, Garner E, Pruden A, et al. DeepARG: a deep learning approach for predicting antibiotic resistance genes from metagenomic data. Microbiome 2018;6:23. 29391044 10.1186/s40168-018-0401-z PMC5796597

[17] Li Y, Xu Z, Han W, et al. HMD-ARG: hierarchical multi-task deep learning for annotating antibiotic resistance genes. Microbiome 2021;9:40. 33557954 10.1186/s40168-021-01002-3 PMC7871585

[18] Sharma D, Xu W. phyLoSTM: a novel deep learning model on disease prediction from longitudinal microbiome data. Bioinformatics 2021;37:3707–14. 10.1093/bioinformatics/btab482 34213529

[19] Chen X, Liu L, Zhang W, et al. Human host status inference from temporal microbiome changes via recurrent neural networks. Brief Bioinform 2021;22:bbab223. 10.1093/bib/bbab223 34151933

[20] After the Integrative Human Microbiome Project . What's next for the microbiome community? Nature 2019;569:599. 10.1038/d41586-019-01674-w 31142868

[21] Proctor LM, Creasy HH, Fettweis JM, et al. The Integrative Human Microbiome Project. Nature 2019;569:641–8. 31142853 10.1038/s41586-019-1238-8 PMC6784865

[22] Thompson LR, Sanders JG, McDonald D, et al. A communal catalogue reveals Earth's multiscale microbial diversity. Nature 2017;551:457–63. 29088705 10.1038/nature24621 PMC6192678

[23] Sunagawa S, Coelho LP, Chaffron S, et al. Ocean plankton. Structure and function of the global ocean microbiome. Science 2015;348:1261359. 25999513 10.1126/science.1261359

[24] Buchfink B, Xie C, Huson DH. Fast and sensitive protein alignment using DIAMOND. Nat Methods 2015;12:59–60. 25402007 10.1038/nmeth.3176

[25] Blin K, Shaw S, Kloosterman AM, et al. antiSMASH 6.0: improving cluster detection and comparison capabilities. Nucleic Acids Res 2021;49:W29–35. 33978755 10.1093/nar/gkab335 PMC8262755

[26] Cimermancic P, Medema Marnix H, Claesen J, et al. Insights into secondary metabolism from a global analysis of prokaryotic biosynthetic gene clusters. Cell 2014;158:412–21. 25036635 10.1016/j.cell.2014.06.034 PMC4123684

[27] Hannigan GD, Prihoda D, Palicka A, et al. A deep learning genome-mining strategy for biosynthetic gene cluster prediction. Nucleic Acids Res 2019;47:e110–0. 31400112 10.1093/nar/gkz654 PMC6765103

[28] Gruber N, Galloway JN. An Earth-system perspective of the global nitrogen cycle. Nature 2008;451:293–6. 18202647 10.1038/nature06592

[29] Yarwood SA . The role of wetland microorganisms in plant-litter decomposition and soil organic matter formation: a critical review. FEMS Microbiol Ecol 2018;94:fiy175. 10.1093/femsec/fiy175 30169564

[30] Helmink BA, Khan MAW, Hermann A, et al. The microbiome, cancer, and cancer therapy. Nat Med 2019;25:377–88. 30842679 10.1038/s41591-019-0377-7

[31] Cheng M, Ning K. Stereotypes about enterotype: the old and new ideas. Genomics Proteomics Bioinformatics 2019;17:4–12. 31026581 10.1016/j.gpb.2018.02.004 PMC6521238

[32] Arumugam M, Raes J, Pelletier E, et al. Enterotypes of the human gut microbiome. Nature 2011;473:174–80. 21508958 10.1038/nature09944 PMC3728647

[33] Truong DT, Tett A, Pasolli E, et al. Microbial strain-level population structure and genetic diversity from metagenomes. Genome Res 2017;27:626–38. 28167665 10.1101/gr.216242.116 PMC5378180

[34] Luo C, Knight R, Siljander H, et al. ConStrains identifies microbial strains in metagenomic datasets. Nat Biotechnol 2015;33:1045–52. 26344404 10.1038/nbt.3319 PMC4676274

[35] Metcalf Jessica L, Xu Zhenjiang Z, Weiss S, et al. Microbial community assembly and metabolic function during mammalian corpse decomposition. Science 2016;351:158–62. 26657285 10.1126/science.aad2646

[36] Li Y, Huang C, Ding L, et al. Deep learning in bioinformatics: introduction, application, and perspective in the big data era. Methods 2019;166:4–21. 31022451 10.1016/j.ymeth.2019.04.008

[37] Libbrecht MW, Noble WS. Machine learning applications in genetics and genomics. Nat Rev Genet 2015;16:321–32. 25948244 10.1038/nrg3920 PMC5204302

[38] Tang B, Pan Z, Yin K, et al. Recent advances of deep learning in bioinformatics and computational biology. Front Genet 2019;10:214. 30972100 10.3389/fgene.2019.00214 PMC6443823

[39] Zou J, Huss M, Abid A, et al. A primer on deep learning in genomics. Nat Genet 2019;51:12–8. 30478442 10.1038/s41588-018-0295-5 PMC11180539

[40] Sunagawa S, Acinas SG, Bork P, et al. Tara Oceans: towards global ocean ecosystems biology. Nat Rev Microbiol 2020;18:428–45. 32398798 10.1038/s41579-020-0364-5

[41] Li J, Jia H, Cai X, et al. An integrated catalog of reference genes in the human gut microbiome. Nat Biotechnol 2014;32:834–41. 24997786 10.1038/nbt.2942

[42] Belilla J, Moreira D, Jardillier L, et al. Hyperdiverse archaea near life limits at the polyextreme geothermal Dallol area. Nature Ecol Evol 2019;3:1552–61. 31666740 10.1038/s41559-019-1005-0 PMC6837875

[43] Yue Y, Shao T, Long X, et al. Microbiome structure and function in rhizosphere of Jerusalem artichoke grown in saline land. Sci Total Environ 2020;724:138259. 32247981 10.1016/j.scitotenv.2020.138259

[44] Korzhenkov AA, Toshchakov SV, Bargiela R, et al. Archaea dominate the microbial community in an ecosystem with low-to-moderate temperature and extreme acidity. Microbiome 2019;7:11. 30691532 10.1186/s40168-019-0623-8 PMC6350386

[45] Wang Y, Feng X, Natarajan VP, et al. Diverse anaerobic methane- and multi-carbon alkane-metabolizing archaea coexist and show activity in Guaymas Basin hydrothermal sediment. Environ Microbiol 2019;21:1344–55. 30790413 10.1111/1462-2920.14568

[46] Simmonds P, Adams MJ, Benkő M, et al. Virus taxonomy in the age of metagenomics. Nat Rev Microbiol 2017;15:161–8. 28134265 10.1038/nrmicro.2016.177

[47] Miao W, Song L, Ba S, et al. Protist 10,000 Genomes Project. Innovation 2020;1:100058. 10.1016/j.xinn.2020.100058 PMC8456420 34557722

[48] Bäckhed F, Roswall J, Peng Y, et al. Dynamics and stabilization of the human gut microbiome during the first year of life. Cell Host Microbe 2015;17:690–703. 25974306 10.1016/j.chom.2015.04.004

[49] Claesson MJ, Jeffery IB, Conde S, et al. Gut microbiota composition correlates with diet and health in the elderly. Nature 2012;488:178–84. 22797518 10.1038/nature11319

[50] David LA, Maurice CF, Carmody RN, et al. Diet rapidly and reproducibly alters the human gut microbiome. Nature 2014;505:559–63. 24336217 10.1038/nature12820 PMC3957428

[51] Faith Jeremiah J, Guruge Janaki L, Charbonneau M, et al. The long-term stability of the human gut microbiota. Science 2013;341:1237439. 23828941 10.1126/science.1237439 PMC3791589

[52] Wu Gary D, Chen J, Hoffmann C, et al. Linking long-term dietary patterns with gut microbial enterotypes. Science 2011;334:105–8. 21885731 10.1126/science.1208344 PMC3368382

[53] Sonnenburg JL, Bäckhed F. Diet–microbiota interactions as moderators of human metabolism. Nature 2016;535:56–64. 27383980 10.1038/nature18846 PMC5991619

[54] Budden KF, Shukla SD, Rehman SF, et al. Functional effects of the microbiota in chronic respiratory disease. Lancet Respir Med 2019;7:907–20. 30975495 10.1016/S2213-2600(18)30510-1

[55] Ni J, Wu GD, Albenberg L, et al. Gut microbiota and IBD: causation or correlation? Nat Rev Gastroenterol Hepatol 2017;14:573–84. 28743984 10.1038/nrgastro.2017.88 PMC5880536

[56] Zhao Y, Wang C-C, Chen X. Microbes and complex diseases: from experimental results to computational models. Brief Bioinform 2021;22:bbaa158. 32766753 10.1093/bib/bbaa158

[57] Gupta VK, Kim M, Bakshi U, et al. A predictive index for health status using species-level gut microbiome profiling. Nat Commun 2020;11:4635. 32934239 10.1038/s41467-020-18476-8 PMC7492273

[58] Wang Y, LêCao K-A. Managing batch effects in microbiome data. Brief Bioinform 2020;21:1954–70. 31776547 10.1093/bib/bbz105

[59] Hall AB, Tolonen AC, Xavier RJ. Human genetic variation and the gut microbiome in disease. Nat Rev Genet 2017;18:690–9. 28824167 10.1038/nrg.2017.63

[60] Glassner KL, Abraham BP, Quigley EMM. The microbiome and inflammatory bowel disease. J Allergy Clin Immunol 2020;145:16–27. 31910984 10.1016/j.jaci.2019.11.003

[61] Fonseca V, Libin PJK, Theys K, et al. A computational method for the identification of dengue, Zika and chikungunya virus species and genotypes. PLoS Negl Trop Dis 2019;13:e0007231–1. 31067235 10.1371/journal.pntd.0007231 PMC6527240

[62] Chen X, Huang Y-A, You Z-H, et al. A novel approach based on KATZ measure to predict associations of human microbiota with non-infectious diseases. Bioinformatics 2017;33:733–9. 28025197 10.1093/bioinformatics/btw715

[63] Huang Z-A, Chen X, Zhu Z, et al. PBHMDA: path-based human microbe-disease association prediction. Front Microbiol 2017;8:233. 28275370 10.3389/fmicb.2017.00233 PMC5319991

