# Supplementary material for: Ontology-aware neural network: a general framework for pattern mining from microbiome data
Source: Brief Bioinform. 2022 Jan 29;23(2):bbac005. doi: 10.1093/bib/bbac005 (PMC8921649; doi:10.1093/bib/bbac005)
Supplement: Supplementary_Table_S1_bbac005 [file supplementary_table_s1_bbac005.docx]

**Supplementary Table S1. Comparison of ONN method and other standard methods for antibiotic resistance gene prediction.**

| **Method** | **Algorithm** | **Accuracy (%)** | **Time usage (s) ^a^** | **Memory usage (GB) ^b^** | **Ref** |
| --- | --- | --- | --- | --- | --- |
| Diamond | Sequence alignment | 38.5 | 2 | 0.1 | [24] |
| DeepARG | DNN | 49.4 | 25 | 0.6 | [16] |
| ONN4ARG | ONN | 93.0 | 10 ^c^ | 2.0 | [13] |

*Note*: The ONN model of ONN4ARG is built based on CARD version 3.0.3 [1]. When we compared the latest CARD version 3.1.4 to the previous version 3.0.3, we discovered 2,281 new ARGs. The 2,281 ARGs were then clustered into 312 clusters with a 90% sequence identity, and the 312 representative ARGs were used as the testing dataset. For Diamond, we searched CARD version 3.0.3 with the testing dataset. For DeepARG, we used the DeepARG program (default parameters) to predict the testing dataset. For ONN4ARG, we used the ONN4ARG program (default parameters) to predict the testing dataset. DNN, deep neural network; ONN, ontology-aware neural network; ^a^, running on a Linux platform with 20 cores; ^b^, maximum memory usage when programs running; ^c^, query time only (excluding training time).
