# Supplementary material for: Ontology-aware neural network: a general framework for pattern mining from microbiome data
Source: Brief Bioinform. 2022 Jan 29;23(2):bbac005. doi: 10.1093/bib/bbac005 (PMC8921649; doi:10.1093/bib/bbac005)
Supplement: Supplementary_Table_S2_bbac005 [file supplementary_table_s2_bbac005.docx]

**Supplementary Table S2. Comparison of ONN method and other standard methods for microbial source tracking.**

| **Method** | **Algorithm** | **AUROC (%)** | **Time usage (s) ^a^** | **Memory usage (GB) ^b^** | **Ref** |
| --- | --- | --- | --- | --- | --- |
| SourceTracker | Bayesian | 93.7 | 821,066 | 1.1 | [5] |
| FEAST | EM | 96.0 | 21,876 | 3.0 | [6] |
| ONN4MST | ONN | 98.0 | < 1 ^c^ | 7.6 | [14] |

*Note*: Evaluation was performed on the standard dataset from the published study by Shenhav et al. [6], and download links are provided at **Supplementary Table S3**. EM, Expectation-Maximization; ONN, ontology-aware neural network; ^a^, running on a Linux platform with 20 cores; ^b^, maximum memory usage when programs running; ^c^, query time only (excluding training time).
